# Sugammadex for Neuromuscular Blockade Reversal: A Narrative Review

**DOI:** 10.3390/jcm14124128

**Published:** 2025-06-11

**Authors:** Sapna Ravindranath, Kevin Backfish-White, John Wolfe, Yatish S. Ranganath

**Affiliations:** Department of Anesthesia, Indiana University School of Medicine, Indianapolis, IN 46202, USA; saravi@iu.edu (S.R.); kmbackfi@iu.edu (K.B.-W.); jwolfe@iu.edu (J.W.)

**Keywords:** sugammadex, neostigmine, neuromuscular blockade reversal, rocuronium, Vecuronuim, neuromuscular monitoring, residual paralysis

## Abstract

**Sugammadex** represents a significant advancement in neuromuscular blockade management, enabling rapid, predictable, and highly effective reversal of steroidal neuromuscular blockers such as rocuronium and vecuronium. This review critically examines recent advances in sugammadex research, particularly over the last decade, detailing its pharmacological profile, clinical efficacy, and safety compared to traditional reversal agents, like neostigmine. Its expanding clinical applications across operating rooms, critical care units, and emergency medicine are discussed, emphasizing dosing recommendations and clinical utility in special patient populations, including individuals with renal impairment, pediatric, obstetric, and obese patients. Economic considerations are explored, highlighting sugammadex’s cost-effectiveness through reduced postoperative complications and enhanced operational efficiency, despite higher initial costs. Finally, the review outlines ongoing research directions, including emerging reversal agents, advanced neuromuscular monitoring technologies, and potential future clinical applications, underscoring sugammadex’s evolving role in improving patient safety and anesthetic practice.

## 1. Introduction

Neuromuscular blocking agents (NMBAs) are essential in modern medical and anesthetic practice, facilitating critical procedures, like endotracheal intubation, optimizing surgical conditions, and assisting mechanical ventilation in both operating rooms and critical care units [1,2]. These agents are crucial for maintaining a stable surgical field by preventing involuntary muscle movements, crucial for the precision and safety required in delicate procedures. However, the use of NMBAs is not without risks; residual blockade can result in significant respiratory complications, impaired airway reflexes, and delayed recovery, presenting clinical management challenges [1,3]. Traditional anticholinesterase reversal agents, notably neostigmine, while commonly employed, are limited by unpredictable efficacy, prolonged reversal times, and undesirable parasympathetic side effects [4,5]. In addition, to counteract parasympathetic effects beyond the neuromuscular junction, neostigmine is routinely co-administered with anticholinergic agents, which may in turn cause adverse effects, such as urinary retention, delayed gastrointestinal recovery, or tachycardia [6,7].

In this context, sugammadex represents a significant innovation by uniquely and rapidly encapsulating and inactivating steroidal neuromuscular blockers, such as rocuronium and vecuronium [8]. This mechanism enables rapid and highly reliable reversal of neuromuscular blockade and has been shown to substantially reduce the risk of postoperative residual paralysis in most patients. Since its initial approval in Europe in 2008 and subsequent approval in the United States in 2015, clinical adoption of sugammadex has rapidly expanded, supported by robust evidence demonstrating its superior efficacy and safety [9,10]. Nevertheless, complete and sustained recovery is not assured in all clinical scenarios. Residual or recurrent paralysis has been documented, especially in cardiac surgery patients and in cases where fixed dosing or qualitative monitoring was used instead of quantitative neuromuscular assessment [11,12,13]. These observations emphasize the need for individualized dosing strategies and routine incorporation of quantitative neuromuscular monitoring—even when using sugammadex.

Despite clear clinical advantages, discussions regarding sugammadex’s optimal use persist, particularly concerning cost-effectiveness and its role in managing specific patient populations, including those with renal impairment, obesity, and in obstetric or pediatric settings [5]. This review critically examines recent advances in sugammadex research, exploring its pharmacological properties, clinical efficacy, safety profile, economic impact, and operational benefits. It also addresses special considerations for targeted patient groups and evaluates potential future developments, guiding anesthesiologists and healthcare decision-makers towards optimizing patient outcomes through evidence-based utilization of this drug. This narrative review is based on literature identified through PubMed, Embase, and reference tracking from relevant articles published between 2010 and early 2025, with emphasis on studies from the last decade. Selection prioritized clinical relevance, focusing on systematic reviews, meta-analyses, and large or high-impact clinical studies.

## 2. Pharmacology

Sugammadex, a modified gamma-cyclodextrin, possesses a unique molecular structure characterized by a lipophilic core and hydrophilic periphery, enabling effective encapsulation of steroidal NMBAs, such as rocuronium and vecuronium, into stable, water-soluble complexes [14]. This encapsulation rapidly reverses neuromuscular blockade by efficiently displacing NMBAs from nicotinic receptors. Sugammadex synthesis involves nucleophilic substitution between 6-per-deoxy-6-per-halo-γ-cyclodextrin and 3-mercaptopropionic acid, with rigorous monitoring to manage potential impurities using advanced analytical methods [15].

**Rapid Action:** Sugammadex binds tightly to rocuronium and vecuronium in a 1:1 molar ratio, rapidly forming an inactive complex swiftly eliminated from plasma [16]. Its pharmacokinetics remain linear across intravenous doses of 1–16 mg/kg, with higher doses providing faster reversal [8]. The volume of distribution at steady state is approximately 11–14 L, indicating limited distribution beyond plasma. Sugammadex exhibits minimal binding to plasma proteins or erythrocytes and undergoes negligible metabolism, being excreted nearly entirely unchanged [8].

**Elimination:** Sugammadex elimination predominantly occurs via renal excretion, with approximately 90% of the administered dose excreted unchanged in urine within 24 h [17]. In patients with normal renal function, the elimination half-life is approximately 2 h, and plasma clearance averages around 88 mL/min [8]. Pharmacokinetics remain linear even at high doses; however, clearance and exposure are notably influenced by renal function. Mild renal impairment moderately prolongs elimination half-life (~4 h), while moderate and severe impairment extend it to approximately 6 and 19 h, respectively, markedly increasing systemic exposure (renal impairment discussed separately in detail) [18].

**Figure 1:**
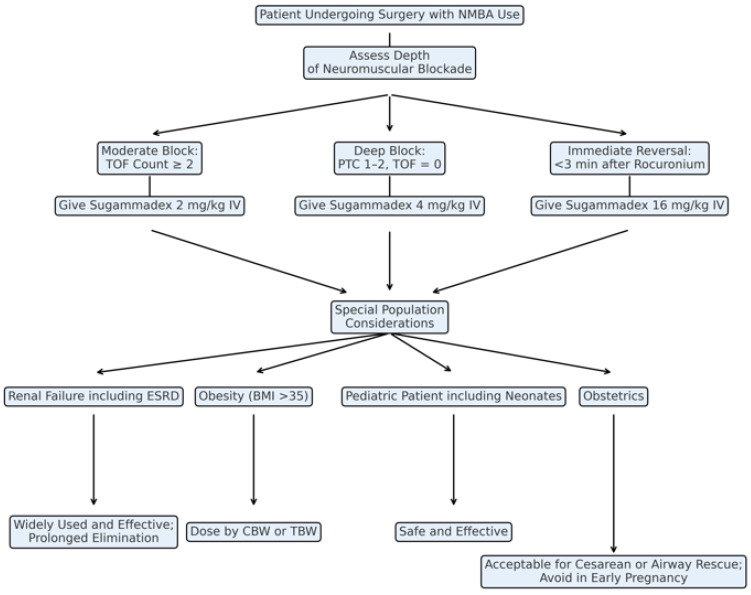
**Clinical Decision Tree for Sugammadex Dosing and Use in Diverse Populations.** NMBA = Neuromuscular Blocking Agent; TOF = Train-of-Four; PTC = Post-Tetanic Count; ESRD = End-Stage Renal Disease; CBW = Corrected Body Weight; TBW = Total Body Weight; BMI = Body Mass Index. Studies evaluating sugammadex use in patients with **renal failure** [19,20,21,22,23,24,25,26], **obesity** [27,28,29,30,31,32,33,34], **pediatrics** [35,36,37,38,39,40], and **obstetrics** [41,42,43,44,45].

**Dosing and Depth of Neuromuscular Blockade** [8] **(Figure 1):** Sugammadex dosage guidelines vary based on the depth of neuromuscular blockade at reversal, categorized clinically into moderate, deep, and immediate reversal:

Moderate Neuromuscular Blockade: Moderate blockade is identified by the reappearance of the second twitch (T2) during train-of-four (TOF) stimulation, indicating partial recovery of neuromuscular function. At this stage, sugammadex administered at 2 mg/kg consistently provides rapid recovery to a TOF ratio ≥ 0.9 within approximately 2–3 min, which is significantly faster than the 10–15 min typically required with cholinesterase inhibitors, such as neostigmine (0.05 mg/kg) [1,10,46].

Deep Neuromuscular Blockade: Deep blockade represents profound suppression of neuromuscular activity, characterized by a post-tetanic count (PTC) of 1–2 with absent TOF twitches. At this depth, sugammadex at 4 mg/kg delivers rapid and reliable reversal. Clinical trials consistently demonstrate significantly faster recovery to a TOF ratio ≥ 0.9 (approximately 3–4 min; neostigmine is not recommended for deep blockade reversal and may take up to 48 min if used) compared to neostigmine or placebo [1,10], reducing variability in recovery, enhancing predictability, and facilitating timely extubation and efficient operating room turnover.

Immediate Reversal: Immediate reversal pertains to rapid antagonism of profound neuromuscular blockade induced by rocuronium shortly after administration (around 3 min post-injection, near peak blockade). This urgent clinical scenario requires a high sugammadex dose of 16 mg/kg. Clinical studies confirm sugammadex at this dose achieves significantly quicker recovery of neuromuscular function (T1 twitch ≥ 10% of baseline) compared to spontaneous recovery from succinylcholine-induced blockade (mean ~4 min versus ~7 min), uniquely allowing anesthesiologists to rapidly reverse profound blockade immediately after induction.

**Dosage Adjustments:** Sugammadex dosing typically relies on total body weight [8]. In mild-to-moderate renal impairment, dose adjustments are generally unnecessary; however, severe impairment significantly prolongs sugammadex elimination, warranting cautious use [18,19]. Clearance decreases by approximately 50% in elderly patients (≥75 years); although dose adjustments typically are not required in elderly patients with normal organ function [47]. Additionally, sugammadex pharmacokinetics do not substantially differ based on hepatic impairment or ethnicity, facilitating consistent dosing guidelines across diverse adult patient populations [17,48]. For morbidly obese patients, dosing based on corrected body weight may be considered, as discussed separately [27].

## 3. Clinical Applications

Sugammadex provides rapid and reliable reversal of amino-steroid NMBAs in diverse clinical settings, extending beyond routine anesthesia into emergency medicine and critical care scenarios.

**Operating Room (OR):** Sugammadex significantly enhances patient safety and surgical efficiency by rapidly reversing moderate and deep neuromuscular blockade. Deep blockade is increasingly favored during laparoscopic and robotic procedures to optimize surgical visualization [49,50]. Sugammadex consistently achieves complete reversal of deep blockade within 2–4 min, substantially reducing residual paralysis risks and associated respiratory complications [10].

**Emergency Department (ED):** Sugammadex is particularly valuable in rapid sequence intubation (RSI), where high dose rocuronium (1.2 mg/kg) often replaces succinylcholine due to contraindications such as hyperkalemia, burns, or stroke. Post-intubation scenarios requiring immediate neurological assessment benefit significantly from sugammadex’s rapid reversal capabilities, clearly outperforming neostigmine [51].

**Intensive Care Unit (ICU) and Post-Anesthesia Care Unit (PACU):** Residual neuromuscular blockade occurs in up to 40–60% of ICU and PACU patients, often due to inadequate neuromuscular monitoring [1,52]. Sugammadex rapidly reverses residual blockade, facilitating earlier extubation, reducing respiratory complications, and improving patient recovery. Its rapid reversal also aids timely neurological assessments or short ICU procedures, such as bronchoscopy [53]. Importantly, sugammadex reversal does not limit subsequent reintubation if high-dose rocuronium is used. Additionally, sugammadex administration in ICU patients intubated with residual paralysis addresses concerns such as patient awareness during paralysis, venous thrombosis, critical illness myopathy, and autonomic disturbances following NMBA use [53].

**Cannot Intubate, Cannot Ventilate (CICV) scenarios:** Sugammadex may be considered following rocuronium-induced CICV situations, although its effectiveness is not guaranteed and remains controversial. The American Society of Anesthesiologists (ASA) highlights limited evidence assessing rocuronium/sugammadex compared to succinylcholine in difficult airway management without specific CICV guidance [54]. A systematic review noted that sugammadex restored spontaneous ventilation in 6 out of 8 cases but led to obstructed breathing requiring surgical intervention in two cases [55]. Thus, sugammadex is a useful adjunct in reversing rocuronium-induced blockade in CICV scenarios but should not replace comprehensive emergency airway management per ASA guidelines, including laryngeal mask airways (LMA) or surgical airway interventions executed in a timely manner.

**Additional Indications:** Sugammadex has proven useful in a variety of distinct clinical settings. It has been used effectively to reverse suspected rocuronium-induced allergic reactions or anaphylaxis, offering clinicians an important perioperative management tool [56]. In patients at risk for postoperative nausea and vomiting (PONV), sugammadex is often preferred over acetylcholinesterase inhibitors due to its avoidance of cholinergic side effects and more favorable recovery profile [57]. In transplant anesthesia, particularly for kidney and liver procedures, sugammadex allows reliable reversal after rapid sequence induction, reducing the risk of prolonged ventilation and improving perioperative management. It is also valuable in neurosurgery and ENT procedures requiring neuromonitoring, where rapid intraoperative reversal supports accurate signal interpretation and surgical precision. During electroconvulsive therapy (ECT), sugammadex offers a safe alternative to succinylcholine, especially in patients with contraindications, such as recent stroke or severe myalgias, enhancing safety and patient comfort [1].

**Considerations in Specific Patient Populations (Figure):** Sugammadex in Obstetrics (Table 1): Sugammadex use is common in non-obstetric patients; however, caution persists in pregnancy due to limited evidence regarding progesterone binding and potential fetal effects. Standard dosing (2–4 mg/kg) remains effective despite pregnancy-induced physiological changes, and sugammadex demonstrates minimal transplacental transfer [41]. Clinical studies have reported its safe use during cesarean delivery, and a separate study of non-obstetric surgery during pregnancy found no increased risk of miscarriage or preterm labor [42,43,44]. SOAP guidelines recommend cautious use during cesarean sections and emergency airways but discourage routine early pregnancy use pending further research. Temporary breastfeeding delay (1 h) post-administration is advised due to limited breastmilk data [45].

Sugammadex in Pediatrics (Table 1): Sugammadex rapidly reverses rocuronium-induced neuromuscular blockade in pediatric patients, including neonates, providing significant advantages over neostigmine [35,36,37,38,39]. Clinical studies consistently report rapid recovery (TOF ratio ≥ 0.9 within 1–3 min), facilitating faster extubation and shorter hospital stays, particularly beneficial for high-risk pediatric populations, such as congenital cardiac surgery patients [35,36,37,39,40]. Safety outcomes favor sugammadex with reduced bradycardia and postoperative nausea/vomiting. Recommended pediatric dosing aligns closely with adults (2 mg/kg moderate, 4 mg/kg deep blockade), demonstrating consistent safety and efficacy.

Sugammadex in Renal Failure (Table 2): Sugammadex elimination primarily occurs renally, raising concerns about prolonged exposure in severe impairment. Renal dysfunction significantly prolongs elimination half-life, extending it to approximately 4, 6, and 19 h for mild, moderate, and severe impairment, respectively, with drug exposure increasing up to 5.4-fold in severe cases [18]. Sugammadex-rocuronium complexes can remain detectable for up to 7 days, ref. [20] though high-flux dialysis removes approximately 69% within 6 h [21].

Sugammadex remains efficacious in chronic kidney disease and ESRD, reliably reversing blockade slightly slower (~1–3 min delay) [20,22]. Safety remains favorable, reducing pulmonary complications compared to neostigmine and showing no adverse kidney transplant outcomes or increased mortality [23,24,25,26]. Recent multicenter data confirm widespread use and efficacy, although higher-dose safety requires further research [19].

Sugammadex in Obesity (Table 2): Sugammadex reliably reverses rocuronium blockade in obese patients, demonstrating superior reversal speed and postoperative outcomes compared to neostigmine [28,29,30]. Total body weight (TBW) dosing reverses blockade faster than ideal body weight (IBW), without increased adverse effects. Corrected body weight (CBW) achieves comparable efficacy to TBW, potentially reducing medication use [27]. CBW is calculated as: IBW + 0.4 × (TBW–IBW), where IBW is estimated using the Devine formula (for men: 50 + 2.3 × [height in inches–60]; for women: 45.5 + 2.3 × [height in inches–60]). Meta-analyses confirm reduced residual paralysis, nausea/vomiting, and cardiovascular complications with sugammadex versus neostigmine [28,30]. Current evidence supports TBW or CBW dosing for optimal efficacy, discouraging IBW-based dosing [27].

## 4. Efficacy and Safety

**Sugammadex vs. Neostigmine in the General Population:** Table 3 consolidates numerous randomized controlled trials and meta-analyses assessing the efficacy and safety of sugammadex compared to neostigmine in the general surgical population. These studies consistently demonstrate sugammadex’s superior performance in rapidly reversing neuromuscular blockade and significantly reducing residual paralysis [10,60,61,62]. However, evidence regarding postoperative nausea and vomiting (PONV) remains mixed. Although earlier systematic reviews, including the Cochrane analysis and ASA Task Force report [1,10], found no clear advantage for sugammadex and rated the evidence quality as low—citing limited patient numbers, wide confidence intervals, and variability across anesthetic techniques—a recent, larger meta-analysis (*n* = 5455) identified a modest but statistically significant reduction in PONV, especially in PACU settings and with volatile anesthetics [57]. These mixed results suggest a potential benefit in certain clinical contexts, yet caution remains warranted when generalizing this finding across broader anesthetic practices. Additionally, the large multicenter retrospective STRONGER trial by Kheterpal et al., involving 45,712 patients, further reinforces sugammadex’s effectiveness in reducing postoperative pulmonary complications (PPCs) [62].

Additional randomized controlled trials by Brueckmann et al. and Cheong et al. provide further evidence of sugammadex’s enhanced operational efficiency and safety [60,61]. Meta-analyses by Wang et al. and Carron et al. corroborate these findings, noting reduced incidences of PPCs and respiratory events [63,65]. Systematic reviews and meta-analyses (SRMAs) emphasize specific postoperative recovery aspects, clearly demonstrating sugammadex’s effectiveness in decreasing PONV [57] and PPCs [63,67,68,69,70]. An SRMA utilizing the Assess Respiratory Risk in Surgical Patients in Catalonia (ARISCAT) index highlights sugammadex’s efficacy in high-risk populations [70]. Sugammadex also significantly reduces postoperative gastrointestinal dysfunction following gastrointestinal surgeries and effectively prevents residual neuromuscular blockade, thereby enhancing patient safety and reducing pulmonary risks [71,72]. However, further research is required to elucidate sugammadex’s effects on overall mortality, length of hospital stay, and quality of recovery across various patient groups and surgical contexts [69]

**Adverse Effects of Sugammadex:** Sugammadex is generally well-tolerated; however, notable adverse effects include hypersensitivity reactions, bradycardia, respiratory, and hematologic complications.

Hypersensitivity and Anaphylaxis: Anaphylaxis associated with sugammadex is rare but clinically significant. Initial FDA safety data indicated an incidence of approximately 0.3%, with subsequent large observational studies reporting lower rates (0.01–0.039%) [73,74]. Increasing clinical usage suggests a projected incidence of approximately 1 in 6000–14,000 administrations [75]. Risk factors include prior sugammadex exposure—even without previous reactions—high-dose administration (particularly 16 mg/kg), atopic history, severe renal impairment, pediatric patients, and underlying cardiovascular disease [75]. Sugammadex has a slightly higher reported incidence of anaphylaxis than neostigmine, necessitating vigilant recognition and management by anesthesiologists [1].

Severe Bradycardia: Sugammadex may cause severe bradycardia or cardiac arrest, with approximately a 1% incidence across various doses [76]. Risk factors include high doses, pre-existing cardiovascular conditions, and renal dysfunction [76]. Effective management involves perioperative monitoring and prompt treatment with anticholinergics like atropine. According to the 2023 ASA Guidelines, bradycardia incidence is similar between sugammadex and neostigmine when neostigmine is paired with glycopyrrolate, suggesting comparable risks when managed with antimuscarinic agents [1].

Other Adverse Effects [77,78]: • Respiratory Spasms: Reports increasingly suggest potential laryngeal and bronchial spasms with sugammadex, even in patients without underlying respiratory conditions, warranting careful postoperative monitoring. • Hypotension: Non-anaphylactic, non-bradycardic hypotension may occur due to vasodilation, volume redistribution following rapid reversal, interactions with perioperative medications, or patient-specific conditions like hypovolemia, especially at higher doses. • Coagulation Effects: Sugammadex may prolong activated partial thromboplastin time (APTT) and prothrombin time (PT), particularly in patients with significant renal impairment, necessitating cautious monitoring. • Interaction with Hormonal Contraceptives: Sugammadex’s binding affinity for progesterone may decrease hormonal contraceptive effectiveness, similar to missing a contraceptive pill dose. Women should employ an additional non-hormonal contraceptive method for seven days post-administration. Despite minimal hormonal impact reported by Devoy et al. [79], precautionary additional contraceptive measures remain recommended.

## 5. Economic and Operational Impact

Sugammadex has a higher acquisition cost compared to neostigmine/glycopyrrolate (neo/glyco), with estimates ranging from USD 75 to USD 148 per 200 mg vial versus USD 12 to USD 87 for neo/glyco [80]. Although sugammadex incurs higher pharmacy costs, multiple studies indicate potential net economic benefits through improved operating room (OR) efficiency, reduced postoperative complications, and fewer hospital re-admissions (Table 4).

Economic analyses show sugammadex reduces total hospital costs primarily through enhanced perioperative efficiency and decreased complication rates [62,64,81,82,83,84,85]. Azimaraghi et al. demonstrated significant reductions in post-anesthesia care unit length of stay (PACU-LOS) and direct hospital costs with sugammadex, particularly in older, high-risk patients, highlighting notable decreases in PONV [83]. Similarly, Wachtendorf et al. reported lower overall direct hospital costs per case with sugammadex, though cost-effectiveness varied depending on patient risk profiles [82].

Operational efficiency studies reveal that sugammadex could save approximately 18.6 min of anesthesia-controlled time and 12 min of PACU time. Given OR costs averaging USD 21 per minute, these savings could offset sugammadex’s higher initial cost. Hurford et al. (2020) concluded that sugammadex becomes cost-effective when OR time is valued above USD 8.60 per minute [86]. Sugammadex also significantly reduces postoperative PPCs. Kheterpal et al. reported a 30% reduction in PPC risk compared to neostigmine, notably lowering pneumonia and respiratory failure rates [31]. Martinez-Ubieto et al. and Jiang et al. further emphasized substantial cost savings driven by fewer respiratory events [84,85].

Cost-saving strategies, such as aliquoting sugammadex from larger vials and guiding administration with quantitative neuromuscular monitoring, have demonstrated substantial economic benefits [87,88]. These approaches reduce drug waste and allow omission of sugammadex in patients with adequate spontaneous recovery, resulting in a net cost savings of approximately USD 46 per case and estimated annual institutional savings of up to USD 370,000 [87]. Co-administration of sugammadex and neostigmine has been proposed as a potential cost-saving strategy and shows preliminary promise [89], but its efficacy, safety, and economic impact remain unclear and have not been formally evaluated.

However, Lan et al. found clinical benefits without cost justification in Taiwan’s low-cost healthcare setting [90]. Further, routine sugammadex use solely to mitigate PONV lacks economic justification [81]. Additionally, faster reversal does not necessarily equate to shorter PACU stays. A prospective randomized study found no significant reduction in PACU discharge readiness time with sugammadex compared to neostigmine [91]. The operational benefit of sugammadex on PACU throughput appears to depend more on institutional workflow and capacity constraints than on pharmacological factors alone.

**Table 4:** Economic and Operational Impacts of Sugammadex Use.

Study Details	Primary Outcomes and Key Results	Secondary Outcomes and Clinical Implications
Azimaraghi et al., 2023 [83]; Retrospective; N = 29,316	PLOS-ACF reduced by 9.5 min; direct cost ↓ USD 77 (*p* < 0.001).	Older/high-risk: ↓ 18.2 min and USD 176; PONV lower (17.2% vs. 19.6%).
Wachtendorf et al., 2023 [82]; Registry analysis; N = 79,474	Direct cost ↓ 1.3%; total per-case ↓ USD 232 (*p* = 0.002).	Low-risk: USD 1,042 saved; high-risk: USD 620 ↑ (*p* < 0.001).
Kheterpal et al., 2020 [62]; Multicenter; N = 45,712	PPCs: 3.5% vs. 4.8% (Sugammadex vs. Neostigmine); OR = 0.70.	↓ pneumonia, respiratory failure; supports pulmonary benefit.
Togioka et al., 2020 [64]; RCT; N = 200	No PPC diff; ↓ residual blockade with Sugammadex (*p* < 0.001).	↓ 30-day readmissions (*p* = 0.03); may improve outcomes.
Hurford et al., 2020 [81]; Cost model (US)	Cost-effective if OR time ≥ USD 8.60/min.	Favored in high-risk or efficiency-driven scenarios.
Martinez-Ubieto et al., 2021 [84]; Spain; N ≈ 537,931	Net savings €57.1 M/year with sugammadex.	Fewer complications; system-wide cost offsets.
Jiang et al., 2021 [85]; US model; N = 100,000	↓ PPCs → net savings ~USD 3.08M (~USD 309/procedure).	Fewer complications drive economic benefit.
Lan et al., 2023 [90]; Taiwan; N = 1784	Faster recovery; ↓ bradycardia (*p* < 0.001).	Higher cost not justified in low-cost setting.

**Legend**: PLOS-ACF = Postoperative Length of Stay–Anesthesia Care Finish; PPCs = Postoperative Pulmonary Complications; RCT = Randomized Controlled Trial; OR = Operating Room; USD = United States Dollar; € = Euro.

Calculating the net economic impact of substituting sugammadex for neo/glyco is complex. While pharmacy cost differences are straightforward, broader implications, including operational efficiencies, reduced adverse events, and decreased PONV rates, must also be evaluated. Economic perspectives vary significantly between reimbursement models. In fee-for-service systems, cheaper reversal agents, like neo/glycol, may be preferred since institutions separately bill prolonged OR time and complications, externalizing these costs. Conversely, in bundled-payment systems, hospitals receive fixed reimbursements per procedure, and the higher initial pharmacy cost of sugammadex can be justified by reducing preventable complications and operational inefficiencies, thus optimizing overall financial outcomes. Overall, although sugammadex remains costlier than neo/glyco, its potential to enhance patient safety and hospital efficiency may provide a favorable cost/benefit ratio in certain surgical settings, particularly under bundled-payment reimbursement models. For patients at high risk for postoperative pulmonary complications or in contexts where improved OR efficiency translates directly into increased productivity, sugammadex’s economic impact may be highly advantageous.

## 6. Future Directions

Ongoing research continues to explore and expand the safety, efficacy, and clinical applicability of sugammadex and neuromuscular blockade reversal. Emerging alternatives to sugammadex, such as adamgammadex—a modified γ-cyclodextrin—are currently under evaluation. Adamgammadex has demonstrated potential in reducing hypersensitivity risk while maintaining comparable efficacy by tightly encapsulating rocuronium and vecuronium [92,93]. Additionally, synthetic molecular containers, such as calabadions and acyclic cucurbit[n]urils, offer broad-spectrum reversal activity against both aminosteroid and benzylisoquinolinium NMBAs, featuring rapid clearance and improved biocompatibility [92,93]. Novel strategies beyond direct NMBA binding, including ClC-1 channel blockers that enhance muscle excitability rather than directly chelating muscle relaxants, are also being explored. These strategies could potentially minimize side effects and extend reversal capabilities [93].

Advancements in neuromuscular monitoring technology, specifically the increased adoption of quantitative neuromuscular monitors, significantly reduce residual paralysis risks, unplanned reintubations, and postoperative pulmonary complications [87]. Recent American Society of Anesthesiologists (ASA) guidelines emphasize routine quantitative monitoring whenever NMBAs are administered, underscoring substantial improvements in patient outcomes associated with educational interventions that promote widespread adoption of this technology [1,94].

Overall, continued development of alternative reversal agents, novel pharmacological approaches, and enhanced monitoring technologies promise to further improve neuromuscular blockade management, broaden clinical utility, and optimize perioperative patient safety and recovery. Additionally, future research should specifically address the safety concerns of sugammadex, particularly hypersensitivity reactions and cardiac effects, while also further exploring its efficacy and safety in special patient populations.

## 7. Conclusions

Sugammadex represents a significant advancement in neuromuscular blockade management, distinctly surpassing traditional anticholinesterase agents by providing rapid, predictable, and complete reversal of steroidal neuromuscular blockers, like rocuronium and vecuronium. It substantially reduces the risks of residual paralysis and related complications, notably decreasing postoperative pulmonary complications, PONV, and postoperative gastric dysfunction. Consequently, sugammadex’s application extends beyond the operating room, benefiting critical care and emergency settings.

Its specific advantages in special populations are notable: Sugammadex significantly improves recovery times and reduces postoperative complications in patients with renal impairment, including those with end-stage renal disease (ESRD), and in pediatric populations, including neonates. For obese patients, dosing sugammadex based on total or corrected body weight is recommended, proving more effective than ideal body weight-based dosing. While sugammadex is cautiously employed during cesarean sections and emergency airway management in obstetric practice, routine use in early pregnancy remains discouraged, despite no demonstrated fetal harm.

The extensive clinical advantages of sugammadex, especially in reducing postoperative complications and shortening recovery times, justify its higher initial cost, particularly in high-risk patient management. Ongoing research continually confirms its safety and efficacy, reinforcing its critical role in contemporary anesthetic practice. As further studies expand its clinical applications and refine usage guidelines, sugammadex is poised to significantly enhance patient outcomes across a broad spectrum of surgical and critical care contexts, profoundly transforming the landscape of neuromuscular blockade management and patient care.

## Figures and Tables

**Table 1:** Sugammadex Use in Obstetrics and Pediatrics.

Author, Year; Study Design	Primary Outcomes	Key Findings and Conclusions
**Obstetrics**		
Stourac et al., 2016 [44]; RCT; N = 240	Sugammadex vs. succinylcholine for Cesarean GA; intubation time, reversal	Noninferior intubation time (+2.9 s); better conditions; lower myalgia (0% vs. 6.7%); no neonatal issues
Kosinová et al., 2017 [43]; RCT; N = 488 women	Apgar scores post Cesarean with Sugammadex	Higher 1-min Apgar < 7 (17.5% vs. 10.3%); resolved by 5 min; no NICU admissions
Noguchi et al., 2023 [42]; Retrospective; N = 124	Non-OB surgery during pregnancy; reversal and complications	No increased miscarriage/preterm birth; effective reversal; safe use in pregnancy
Richardson and Raymond, 2020 [41]; Review	Literature summary through 2020 on OB safety	No OB complications; minimal placental transfer; cautious 1st trimester use
**Pediatrics**		
Ammar et al., 2017 [39]; RCT; N = 60	Sugammadex vs. Neostigmine (2–10 yrs)	Faster TOF ≥ 0.9 (2.5 vs. 12.6 min); less PONV, tachycardia
Franz et al., 2019 [36]; Retrospective; N = 331	Infants < 2 yrs; Sugammadex vs. Neostigmine	Faster reversal (84 vs. 103 min); safe; 2 mg/kg effective
Gaver et al., 2019 [40]; Retrospective; N = 968	0–17 yrs; reversal time, adverse events	Lower bradycardia; faster OR exit (−2.8 min); no hypersensitivity
Li et al., 2021 [37]; RCT; N = 60	Infants < 3 yrs with CHD; reversal and recovery	Faster TOF (3.4 vs. 76.2 min); less atelectasis; shorter stay
Voss et al., 2022 [35]; RCT; N = 276	Children 2–<17 yrs; moderate block	TOF recovery 1.6 vs. 7.5 min; low bradycardia; reliable across ages
Lang et al., 2022 [38]; Meta-analysis; 14 RCTs	Sugammadex vs. Neostigmine in pediatrics	Faster TOF (−10.3 min); fewer PONV/bradycardia; limited infant data

RCT = Randomized Controlled Trial; GA = General Anesthesia; TOF = Train-of-Four ratio; OB = Obstetric; NICU = Neonatal Intensive Care Unit; CHD = Congenital Heart Disease; PONV = Postoperative Nausea and Vomiting; OR = Operating Room; min = minutes.

**Table 2:** Sugammadex in Renal Failure and Obesity.

Author, Year; Study Design	Primary Outcomes	Key Findings and Conclusions
**Renal Failure**		
Panhuizen et al., 2015 [20]; Prospective; N = 67	TOF 0.9 recovery, PK, safety	TOF recovery slower (3.1 vs. 1.9 min); exposure prolonged; no recurarization.
Ono et al., 2018 [24]; Retrospective; N = 99	Reversal efficacy; periop/6-month outcomes	All reversed; no intra-op or long-term complications; graft function preserved.
Adams et al., 2020 [58]; Retrospective; N = 158	Reintubation, delayed extubation	1.9% reintubation, unrelated to NMB; no recurrence.
Paredes et al., 2020 [59]; Retrospective; N = 219	Sugammadex complications; mortality	Low adverse event rate: none linked to sugammadex; 4% mortality unrelated.
Oh et al., 2024 [22]; RCT; N = 49	TOF ≥ 0.9; adverse events	Recovery 3.5 vs. 14.8 min vs. neostigmine; no serious events.
Elkhateb et al., 2025 [19]; Retrospective; N = 243,944	Practice trends in eGFR < 60; ESRD use	Use increased to 95%; ESRD 87%; higher doses common; supports routine use.
**Obesity**		
Abd El-Rahman et al., 2017 [31]; RCT; N = 180	1.5–4 mg/kg IBW for mod block	All reversed; 4 mg/kg fastest; no safety issues.
Elfawy et al., 2019 [32]; RCT; N = 60	IBW vs. ABW vs. AdjBW dosing	ABW fastest; all safe; IBW least effective.
Ornek et al., 2020 [33]; RCT; N = 60	IBW vs. CorrBW vs. TBW	TBW fastest; all tolerated; IBW slowest.
Horrow et al., 2021 [29]; RCT; N = 188	ABW vs. IBW vs. Neostigmine	ABW best; faster than neostigmine; safe profile.
Subramani et al., 2021 [30]; Meta-analysis; N = 386	Sugammadex vs. Neostigmine	Faster reversal; fewer complications; sugammadex preferred.
Liao et al., 2022 [27]; Meta-analysis; N = 444	IBW vs. TBW	TBW faster; IBW underdosed; CorrBW comparable.
Wang et al., 2024 [28]; Meta-analysis; N = 633	Sugammadex vs. Neostigmine	Reduced PORC; faster recovery; favors sugammadex.
Ajetunmobi et al., 2025 [34]; RCT; N = 120	OSA patients; sugammadex vs. neostigmine	Similar outcomes; no clear advantage in OSA group.

**Legend**: RCT = Randomized Controlled Trial; TOF = Train-of-Four ratio; PK = Pharmacokinetics; NMB = Neuromuscular Blockade; IBW = Ideal Body Weight; ABW = Actual Body Weight; TBW = Total Body Weight; CorrBW = Corrected Body Weight; AdjBW = Adjusted Body Weight; OSA = Obstructive Sleep Apnea; PORC = Postoperative Residual Curarization.

**Table 3:** Major Studies and Meta-Analyses Comparing Sugammadex and Neostigmine in the General Population.

Study Details	Primary Outcomes and Key Results	Secondary Outcomes and Clinical Implications
Brueckmann et al., 2015 [60]; RCT; N = 154	Faster OR discharge readiness with Sugammadex (*p* < 0.05).	Improved efficiency; fewer adverse events (RR 0.39, CI 0.11–1.41).
Cheong et al., 2015 [61]; RCT; N = 120	Fewer adverse events with Sugammadex (RR 0.13, CI 0.03–0.55).	Neostigmine combo lowers dose but raises side effect risk.
Carron et al., 2016 [63]; Meta-analysis; 13 RCTs; N = 1384	Sugammadex faster for moderate/deep block (12.9 vs. 48.8 min).	More reliable reversal; fewer residual paralysis and complications.
Hristovska et al., 2017 [10]; Meta-analysis; 41 RCTs; N = 4206	Faster reversal (mod: −10.2 min; deep: −45.8 min).	Lower bradycardia (RR = 0.16), PONV (RR = 0.52), paralysis (RR = 0.40); SAE similar.
Kheterpal et al., 2020 [62]; Retrospective; N = 45,712	Lower PPCs with Sugammadex (3.5% vs. 4.8%).	Fewer pneumonias and respiratory failures; reduced PPC risk.
Togioka et al., 2020 [64]; RCT; N = 197	PPCs similar; residual paralysis lower with Sugammadex.	Improved recovery; potential for fewer readmissions.
Wang et al., 2021 [65]; Meta-analysis; 14 RCTs; N = 1478	Fewer PPCs, mainly respiratory failure (OR 0.62).	Pulmonary benefit driven by fewer ventilatory support needs.
Suleiman et al., 2023 [66]; Retrospective; N = 83,250	No difference in respiratory complications or healthcare use.	Neostigmine non-inferior for general population safety.
Liu et al., 2023 [67]; Meta-analysis; RCTs + obs.	Reduced PPCs: pneumonia, atelectasis, reintubation with Sugammadex.	Supports pulmonary benefit; further trials needed.

**Legend**: RCT = Randomized Controlled Trial; TOF = Train-of-Four; RR = Relative Risk; CI = Confidence Interval; PPCs = Postoperative Pulmonary Complications; PONV = Postoperative Nausea and Vomiting; NNT = Number Needed to Treat; OR = Operating Room; SAE = Serious Adverse Events.
